# Diagnosis of Child Sexual Abuse

**DOI:** 10.3390/jcm13237297

**Published:** 2024-11-30

**Authors:** Roland Csorba, Zeynep Atas Elfrink, Panagiotis Tsikouras

**Affiliations:** 1 Department of Obstetrics and Gynaecology, University of Duisburg-Essen, D-45147 Essen, Germany; zeynep.ataselfrink@uk-essen.de; 2 Unit of Maternal-Fetal-Medicine, Department of Obstetrics and Gynecology, Medical School, Democritus University of Thrake, GR-68100 Alexandroupolis, Greece; ptsikour@med.duth.gr

**Keywords:** child sexual abuse, medical diagnosis, differential diagnosis, forensic documentation

## Abstract

Child sexual abuse (CSA) is a widespread and alarming issue, with an estimated global prevalence of 12–13% (affecting 18% of girls and 8% of boys). Despite its prevalence, many physicians working with children have insufficient knowledge of the medical diagnosis of CSA. This lack of expertise, combined with the sensitive and complex nature of these cases, often hampers proper identification and management. Diagnosing CSA is particularly challenging and requires specialized skills. A majority of children assessed for suspected sexual abuse present with normal genital and anal findings, which complicates the diagnostic process. Barriers such as professional isolation, societal taboos, and the sensitive nature of the subject often result in diagnostic failures. Accurate medical history taking, diagnosis, and documentation of findings are essential for ensuring a precise diagnosis, safeguarding children, and supporting legal proceedings. However, achieving these goals remains elusive without standardized guidelines and adequately trained healthcare professionals. Training of professionals in observing and reporting child sexual abuse is badly needed. This review explores the current state of medical diagnosis in suspected cases of CSA. This article is based on a selective review of pertinent literature retrieved from various databases, including PubMed and the overall index of the Quarterly Update.

## 1. Introduction

Child sexual abuse (CSA) is alarmingly prevalent, affecting 12–13% of children worldwide (18% of girls and 8% of boys) [1]. This is only the tip of the iceberg; the actual prevalence is likely much higher, as only a small fraction of cases become known. Knowledge of the exact number of sexually abused children and the establishment of a systematic approach to address the medical and legal needs of alleged child victims are yet to be developed. Sexual violence is the least documented crime. According to recent knowledge, CSA is similar to adult sexual abuse [2]. Due to the frequency of this topic, its serious health and social consequences, and the role of the media, CSA has become a focal point in medical and criminal law practice [3,4]. This “silent epidemic” claims more lives of young people than diabetes and malignancies combined for the same age group [5]. Physicians who deal with children often have suboptimal knowledge of CSA, including the characteristics of victims and perpetrators, the medical diagnosis and therapy of sexual abuse, and the rehabilitation of victims [6]. Medical professionals working in primary care, clinics, and institutions, as well as parents, guardians, and teachers who interact with children, should consider the possibility of sexual abuse when children present with genital symptoms. Furthermore, they must be aware that these crimes should be diagnosed appropriately, as described below. In this paper, we do not address the definition of CSA or the knowledge of legal regulations and proceedings. Our main goal is to summarize current information related to the diagnosis of CSA in victims. We emphasize the importance of medical diagnosis and a multiprofessional approach to CSA. Additionally, we aim to clarify the utility of physical examinations and their potential benefits for affected children, even though definitive findings that indicate a diagnosis are rare [5]. This article focuses on the history taken from the victim and the parents, beyond the physical findings of abuse, rather than its emotional and psychiatric consequences.

## 2. General Information

### 2.1. Definition

Child sexual abuse is the involvement of children and adolescents in sexual activities that they cannot fully comprehend and to which they cannot consent as a fully equal, self-determining participant, because of their early stage of development. Child sexual abuse is no longer ‘‘another hidden paediatric problem’’ as stated by Kempe in 1978 [7]. Social taboos are violated, and the offending adults exploit the difference of age and power through verbal persuasion and/or physical compulsion. The intent, on the part of adults, to use children for their own sexual stimulation and satisfaction is the central feature of child sexual abuse. The spectrum ranges from non-invasive activities that do not involve any touching of the child (hands-off contact) all the way to rape. Sexual abuse is usually a chronic, complex, and markedly traumatizing occurrence for the victim, frequently perpetrated by family members or other trusted persons in the setting of relationship dependence and strong authority relationships [6]. The abuse is frightening and deeply emotionally disturbing for the victim and brings about a fundamental disturbance of sexual development. It can give rise to profound feelings of guilt and shame, as well as low self-esteem and familial and social isolation. It has a marked, albeit variable, effect on the victim’s mental, emotional, and physical health.

### 2.2. Importance

Child sexual abuse has the potential for long-term medical and mental health consequences. There are also significant economic and societal implications [8]. Women with a history of childhood sexual abuse account for significantly higher primary care and outpatient costs than women without any such history [9]. Child sexual abuse is associated with an increased risk of poor physical and mental health in adulthood. Many studies have highlighted the development of secondary mental health problems, with manifestations such as suicide, depression, post-traumatic stress disorder, eating disorders, and multiple personality disorders, e.g., child sexual abuse accommodation syndrome. The problems that can result from sexual abuse in childhood include a full spectrum of emotional, psychosomatic, self-destructive, and antisocial behaviors [10,11]. Physical health can be affected, as sexually abused children are at greater risk in adulthood for risk-taking behaviors that include smoking, substance abuse, and alcoholism. Psychophysiological consequences include premenstrual syndrome, unexplained gastrointestinal symptoms, chronic pelvic pain, and problems of pregnancy [12]. Media headlines routinely raise public awareness about the issue of sexual victimization of children and adolescents. Child abuse comes to our attention almost exclusively when a personal tragedy happens.

## 3. History Taking

Interacting with children who may be victims of sexual abuse requires time, training, and commitment. The physician must be sympathetic but must also proceed in a rational, scientifically well-founded manner (“cool science for a hot topic”). It is extremely important to take an accurate medical history in a precisely prescribed manner. The majority of abused children have no visible injuries, and medical evidence is often lacking. Even though more than 90% of abused children have no abnormal findings on physical examination, the forensic diagnostic aspect of the examination must not be neglected, because the absence of positive findings can also be forensically relevant [13]. It cannot be emphasized enough that, most of the time, the only evidence during legal proceedings is the anamnesis provided by the victim. Taking the child’s history is a unique process; its recorded form can be used in subsequent legal proceedings. In cases of suspected CSA, parents, caregivers, or teachers typically turn to the pediatrician or family doctor closest to their homes, expecting them to assess whether abuse has occurred. The attitude and knowledge of healthcare professionals who first see and examine CSA victims is extremely important. While primary care professionals (PCPs) may be reluctant to assess CSA, they are well-positioned to play a key and necessary role. Our aim is to provide these healthcare professionals with practical support in this crucial situation. PCPs often lack experience in assessing children when sexual abuse is suspected and may prefer to refer cases to an emergency department or medical expert while reporting concerns to Child Protective Services (CPS) and/or the police. Therefore, PCPs can play an important, albeit limited, role in initially assessing children when CSA is a concern, particularly in history taking and medical diagnosis [14]. However, pediatric PCPs have the advantage of usually having long-term trusting relationships with patients and families, which can be particularly helpful in stressful situations [15]. Children may be more inclined to share sensitive information with healthcare professionals they know and trust [16]. As the suspicion of abuse is extremely upsetting to the whole family, the accompanying parent or guardian may feel uncertain, helpless, and full of fear and anger. It is recommended to question the parent or caregiver and the child separately and then perform the examination together. If this is not possible, the child should be placed on the parent’s lap to minimize eye contact and parental influence. It should be explained that this is not a real gynecological examination, there will be no injections, and there will be no pain. The questions to be asked of the parent or caregiver are listed in Table 1 [14].

What the parent said can later be verified with the victim’s own words, with particular attention to physical symptoms such as dysuria or vaginal discharge, and behavioral symptoms such as changes in sleep, appetite, and mood [17]. The recorded history must include the time, place, and circumstances of the crime, as well as the personality and characteristics of the alleged perpetrator, and the type and nature of the assault. The child’s medical history should cover any previous genital infections, injuries, prior abuse, sexually transmitted diseases, and any psychological or psychiatric conditions.

After speaking with the accompanying person, the victim should be questioned, preferably alone. The child’s initial account is extremely important for possible legal proceedings. A confession that is precisely recorded and in the recommended format can be used without limitation during legal proceedings, minimizing further trauma for the victim. The victim’s account should be documented verbatim, using her/his own words. The victim’s age, mental capacity, behavior, and the behavior of the accompanying person must be taken into consideration.

During the history-taking process, conditions should be created to encourage the victim to speak freely about the circumstances of the abuse. It can be helpful to start the interview with general questions—about school, friends, or hobbies—in order to ease the child’s fear and break the silence. Cognitive abilities should be assessed with general questions, and the child should be reassured that the doctor is there to help: “We are here to make sure you’re healthy and safe”. Effective follow-up questions include “You can tell me; I am the doctor. What would you like to talk about today?” [14,18].

It helps if the doctor explains to the victim that their job is to protect the physical and mental health of children. The child should understand that any unpleasant or unusual event can be reported to the doctor. It is important to ensure that the child can distinguish truth from falsehood, knows the parts of the body, and can describe what happened in an age-appropriate manner. The language used in the conversation should be suitable for the victim’s age, and suggestive questions must be avoided. Open-ended questions should be asked, and prejudice should be avoided. Asking, “Is there anything that bothers you? Do you have any problems?” will typically elicit the response, “No”. It is more effective to ask, “Can you tell me why you are here to see the doctor?” [19]. During history taking, the doctor should appear calm, trustworthy, and helpful. Without trust, a complete anamnesis cannot be achieved. The victim should also be allowed to ask their own questions. Table 2 provides a summary of the questions asked of the victim [14,19,20].

Questions such as “Tell me what happened” or “Can you describe why you’re here today?” are more effective than asking, “Do you know why you’re here?”. Children often have their own terms for body parts, and it is essential to use their language when discussing sensitive topics. Asking questions like, “What do you call the part of your body where you pee?” can help establish clarity. Healthcare providers should avoid suggesting or leading questions, as these can contaminate the child’s account, making it less reliable for legal purposes. A typical open-ended question is, “Please tell me what happened? What happened after that?” Children give more detailed and precise answers to open-ended questions or questions beginning with what, where, who, when, and why, compared to yes–no or forced-choice questions [21,22]. Yes–no questions should be avoided because a reluctant or anxious child will simply answer “no”. They often respond with “no” to questions that are difficult for them to understand. Victims may deny the abuse due to shame, humiliation, or a self-defense mechanism arising from love and respect for the perpetrator [23].

An effective question to start with regarding abuse is “Tell me, what would you like to share with me?” A less useful question is “Do you know why you are here today?” [24]. If abuse is suspected, it helps to first find out the child’s terms for their genitals and anus, as children often use varied names for these body parts. It is helpful to ask, “What do you call the place where pee comes out?” For the question, “Did anybody touch your private parts?” the answer will often be “no”. It is more effective to ask, “Tell me everyone who has touched your private parts”.

Children are not always aware of the correct terminology, so it is less appropriate to ask about possible penetration. The victim may not be able to distinguish whether penetration occurred or if the perpetrator simply touched them. A 2016 study highlights this communication challenge, where only one question was asked: “When you wipe your vagina after peeing, do you wipe inside or just outside your private parts?” Of the 533 responding children, 41% answered that they wiped inside the vagina, 35% said outside, and 23% stated both inside and outside. It is likely that they all only wiped the outside, yet 69% of children under 12 years of age answered that they wiped either inside or both inside and outside [25].

Contrary to popular belief, the majority of child sexual abuse (CSA) cases are chronic and involve multiple occurrences [6]. Due to delayed examinations of CSA victims, it is not expected that children will be able to determine the exact date of the crime. Recognizing child sexual abuse (CSA) is challenging due to the often missing or difficult-to-assess symptoms and the lack of sufficient experience in evaluation. Healthcare professionals working with children often lack adequate knowledge about diagnosing sexual crimes. The aim and timing of the medical examination are summarized in Table 3 and Table 4 [6,26].

Primary care professionals unfamiliar with the appearance of physiological genital changes may often suspect signs of CSA, especially if the child’s history raises concerns. It is important to emphasize that no signs of injury or infection are found in the majority of CSA victims [27]. Genital injuries heal rapidly, and examination findings are often normal in children whose medical assessment is delayed. Additional reasons include non-touching forms of CSA and forms of oral abuse [26]. History taking and the verbal preparation of the child for physical examination take much more time than the physical examination itself, which usually requires no more than a few minutes. In most of the cases, 30–45 min will be needed overall.

## 4. Examination

The physical examination should only be performed after thorough explanation and with the child’s permission. Its main purpose is the assessment of the anogenital area. Because the tissues in this area are capable of rapid and usually complete regeneration, physical injuries caused by abuse become less evident over time; this accounts for the rarity of positive findings.

The gynecological investigation includes a ‘head-to-toe’ physical examination, assessment of sexual development, identification of any injury with special reference to colposcopic appearances of the hymenal membrane and surrounding structures, signs of abuse, diagnosis of a possible pregnancy by a urine test or ultrasound examination, collection of forensic evidence (sperm, saliva, other trace evidence) and evaluation for sexually transmitted infections. Appropriate medical or surgical treatment is provided based on examination findings. All relevant findings are documented and incorporated into a report and distributed in response to official requests.

In essence, the physical examination in cases of suspected sexual abuse consists of inspection of the anogenital region through a variety of examining methods and techniques while the child is suitably positioned: supine, in the knee–chest position, and in the lateral decubitus position [26]. A combination of three standard techniques—labial separation, labial traction, and knee–chest position—increases the yield of positive findings and is also required by the current Adams classification for a finding to be designated as definitive evidence of abuse. All injuries should be meticulously documented [27]. The use of colposcopy is now standard, as it combines the advantages of excellent lighting, magnification, and high-quality documentation. This also aids in the checking of definitive findings and their confirmation by a second examiner (as currently required) and obviates the need for further, repetitive follow-up examinations, which may be emotionally traumatizing [19].

### 4.1. Physiological Genital and Anal Findings

**Figure 1:**
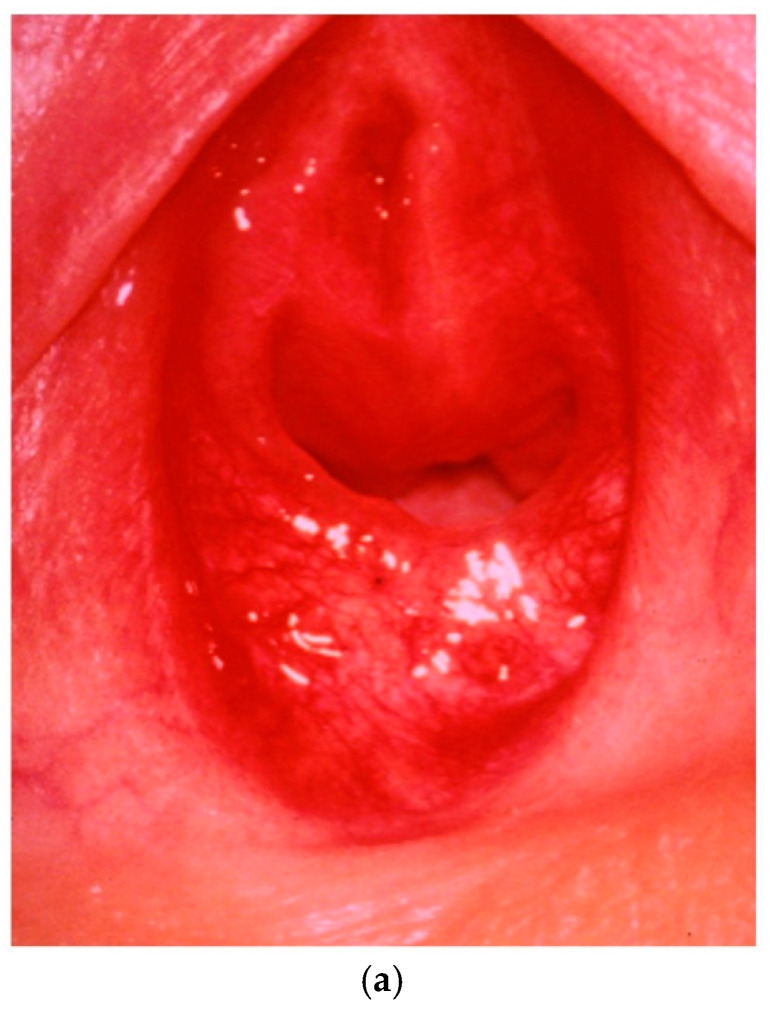

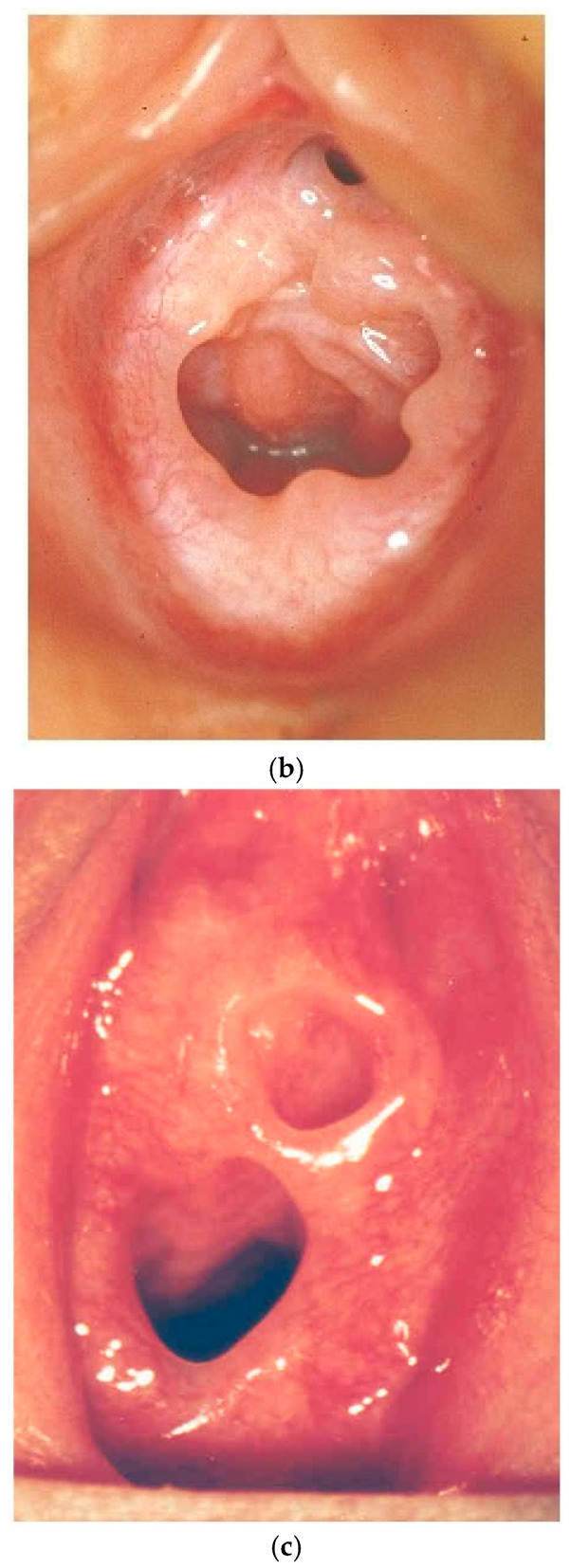

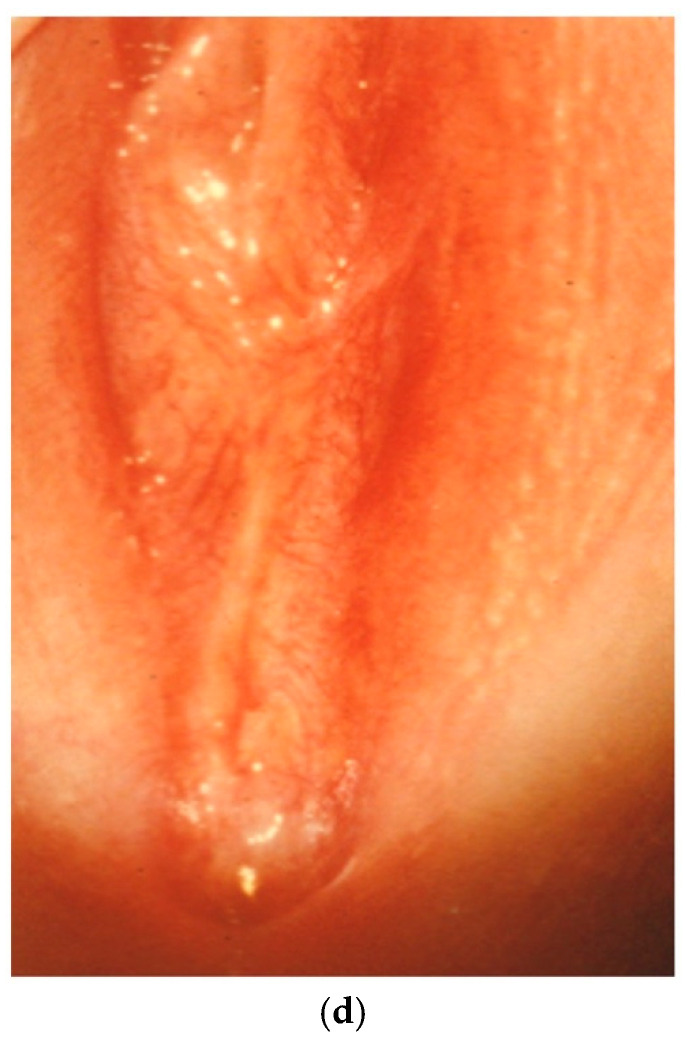
(**a**) Semilunar hymen. (**b**) Cribriform hymen. (**c**) Septate hymen. (**d**) Imperforate hymen.

The appearance of the external genitalia, particularly the hymen, depends on the child’s age, as well as structural and hormonal factors. Different physiological variations are illustrated in Figure 1.

Table 5 and Table 6 summarize the physiological genital and anal findings in children.

**Table 5:** Physiological genital findings in girls [5,19].

Various hymenal configurations: septate, semilunar, microperforate, cribriform, imperforate (Figure 1)
External hymenal ridges
Longitudinal intravaginal ridges
Hymeneal tags
Periurethral bands
Erythema in introitus
Congenital pigmentation
Urethral dilatation
Linea vestibularis: an avascular line in the midline of the fossa navicularis

**Table 6:** Physiological anal findings.

**Figure 2:**
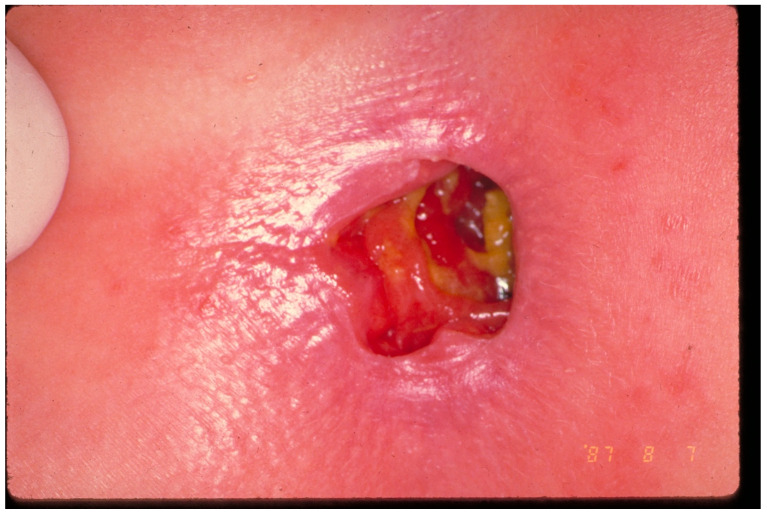
Reflex anal dilatation.

Perianal erythema
Perianal pigmentation
Circular venous engorgement
Perianal polyp-like tags
Diastasis ani (Figure 2)

Many clinical findings that were once misinterpreted as evidence of abuse are now recognized as normal variations. For example, measuring the width of the hymenal opening is now considered obsolete and holds no practical significance. Tampons may widen the hymenal opening but do not cause injury. Similarly, activities such as gymnastics, running, stretching, and masturbation do not cause hymenal injury [27,28]. Every two years, Adams et al. update the most widely accepted classification of examination findings related to CSA, with a simplified version shown in Table 7 [28,29].

### 4.2. Normal Genital Findings Despite Penetration

The medically documented fact that penetrating abuse may not be associated with any abnormal physical findings must be understood by healthcare professionals and government authorities (police, prosecutors), so that the credibility of the victims is not unjustly called into question. The questionable utility of this term in the context of potential sexual abuse is highlighted by a study in which only two (6%) of thirty-six pregnant teenagers showed clear evidence of prior penetration injury, and only four (11%) had suspicious, though not definitive, findings: “‘Normal’ does not mean ‘nothing happened’” [30]. The examination findings of CSA victims are mostly physiological, whether the violence is acute or chronic, with or without penetration.

### 4.3. Anogenital Pathological Findings in Abused Children

The anogenital findings in child sexual abuse vary greatly, depending on the type and frequency of the abuse. They are influenced by the objects used, the degree of violence, the age of the victim, and the intensity of self-defense. The only factors significantly correlated with the diagnosis of findings related to child abuse are pain, vaginal bleeding, and the elapsed time since the last traumatic event [26]. The classification of findings is helpful for assessment, understanding, and interpretation. The three-level Adams classification is widely accepted and serves as the primary guideline for assessing anogenital findings in cases of suspected CSA. Over the past decade, this classification has been continually updated and further developed, most recently in 2023 [31].

**Figure 3:**
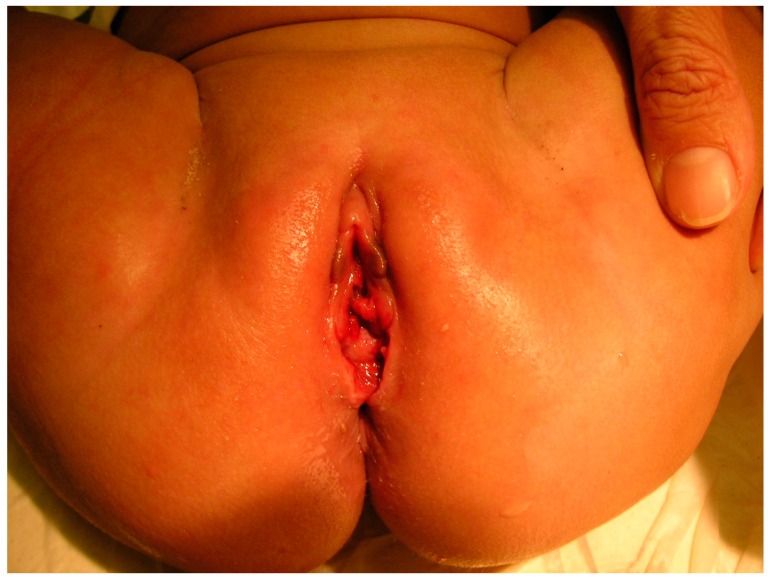
Blunt force trauma in an 18-month-old girl.

The range of examination findings in CSA victims is extremely wide, from erythema of the external genitals to severe penetration injuries (Figure 3) [32].

**Figure 4:**
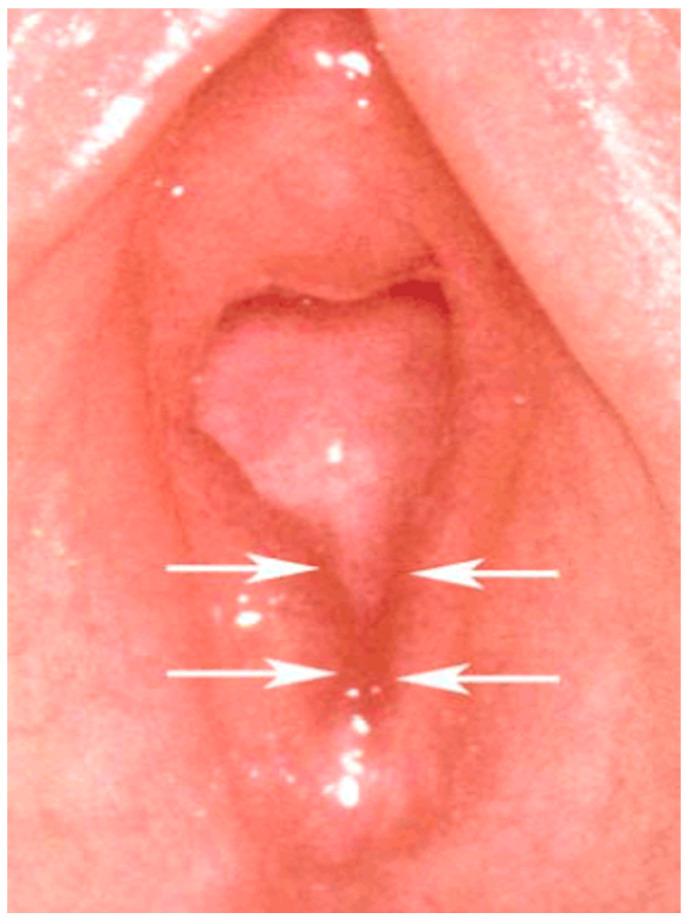
Complete cleft (concavity) at 6 o’clock, an Adams class III finding.

Non-specific symptoms include lower abdominal pain, recurrent dysuria, urinary tract infections, and genital pain and itching. Specific signs include injury and inflammation of the anogenital region, pregnancy, and sexually transmitted diseases that would not otherwise occur. Most findings from abuse are located in the posterior part of the hymen and introitus. An interruption of the peripheral edge of the hymen between the 3 and 9 o’clock positions (with the patient in a supine position) is caused by penetration and can often be seen most clearly in the knee–chest position. As a consequence of such trauma, a V-shaped notch (Figure 4) may appear, which can later develop into a U-shape, called a “concavity” [26]. Hymenal injuries, even in the prepubertal hymen, can heal “ad integrum” [33].

**Figure 5:**
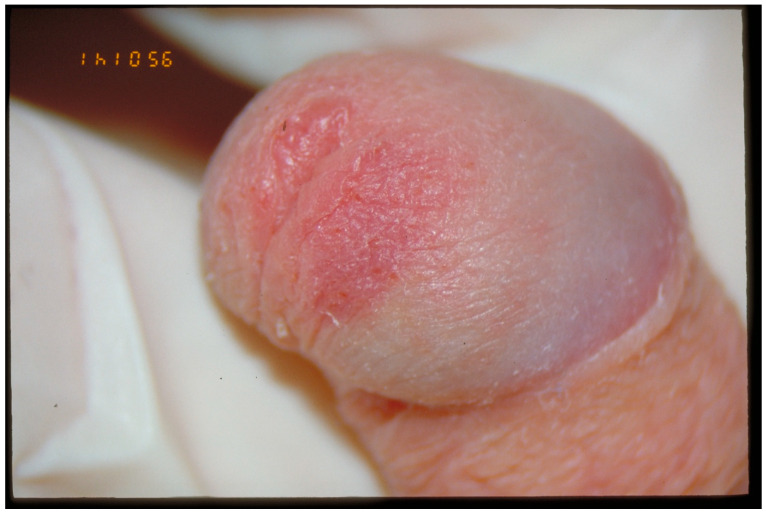
Petechiae and abrasions of the glans penis by a young boy.

Genital injury findings are rare in sexually abused girls (5–10% [5,26]) and even rarer in sexually abused boys (around 1–3%). In boys, injuries may take the form of fissures, abrasions of the penile shaft or glans penis, tears of the frenulum, petechiae, or marks from biting or sucking [34]. Figure 5 shows the penis of a sexually abused boy.

Signs of acute anal abuse include deep perianal tears and hematomas, which are not always clearly visible during a physical examination. Diagnosing internal rectal injuries requires rectoscopy or anoscopy, along with the collection of appropriate forensic trace evidence. The signs of chronic anal abuse remain unclear, and the significance of chronic changes in the anal region is controversial.

One finding, known as “reflex anal dilatation”, can suggest potential—but not definitive—evidence of abuse, particularly if the anal opening widens to more than 2 cm in the absence of stool in the ampulla (Figure 1).

Anal fissures may indicate, but do not necessarily prove, anal penetration [35]. In summary, CSA can be confirmed with certainty in the following cases: pregnancy, sexually transmitted diseases (e.g., Neisseria gonorrhoea, postnatal syphilis), pathological examination findings based on the Adams III classification, or proof of the perpetrator’s DNA on the victim’s body or in the vagina/anus of the victim [26].

## 5. Discussion

The sexual abuse of children is alarmingly prevalent. Data from 39 prevalence studies conducted in 28 countries between 1994 and 2007 reveal that 10–20% of girls and 5–10% of boys are victims of child sexual abuse (CSA) [36]. In the USA, where the reporting of child abuse is mandatory, 60,000 to 80,000 confirmed cases are reported annually, though there has been a downward trend [37]. CSA is a significant and not uncommon problem in Germany, and concerns about CSA can arise in various medical contexts. However, the available data from Germany are limited, and many cases are believed to go unreported. Reliable data on the frequency of CSA subtypes are also sparse. Research shows a lifelong association between sexual victimization in childhood or adolescence and chronic mental and physical illness in adulthood [38].

Examining a child with suspected sexual abuse or anogenital symptoms is a challenge for health care professionals and primary care providers (PCPs). Physicians must also be aware of conditions that can mimic the symptoms of CSA. Recognizing and diagnosing sexual abuse is not easy; it is a complex and multifaceted task for professionals dealing with such cases. The difficulty in assessing examination findings often stems from professional inexperience, isolation, and the fact that sexual violence remains a taboo subject. The majority of abused girls and boys have no visible or diagnosable injuries. Forensic identification of the abuser’s DNA is possible only in exceptional cases, as days or weeks often elapse between the last instance of abuse and the physical examination. If the victim is examined immediately after the event, the likelihood of finding the abuser’s DNA is much higher. DNA traces are rarely found in prepubertal victims and, in exceptional cases, more than 24 h after the event. More forensic attention should be given to the victim’s clothing and bedclothes [39,40]. The German Federal Child-Protection Act specifies the circumstances under which a physician can breach a child’s confidentiality to provide important information to the Youth Welfare Office.

## 6. Conclusions

Health care professionals and PCPs often lack sufficient knowledge about CSA, particularly regarding the recognition and treatment of such crimes. Suspected cases of CSA require a time-consuming diagnostic evaluation, which must be performed with the utmost care and medical expertise. Physicians conducting these evaluations should be experienced in both adolescent gynecology and forensic medicine. If biological evidence needs to be collected, advice should be sought from the responsible forensic medical authorities. Examiners should be familiar with the current state of knowledge regarding the medical findings of CSA and their classification. In 90–95% of cases, only normal findings are revealed during the examination, which seldom leads to a definitive diagnosis or legal resolution.

The diagnosis of sexual abuse is usually based on the victim’s statement, which must be obtained through sympathetic but non-suggestive questioning. Leading questions should be avoided, and health care professionals trained in the psychology of legal testimony should document the child’s answers verbatim. The physical examination itself can have a beneficial therapeutic effect by affirming the child’s bodily integrity and normality, provided it is conducted without coercion. Additionally, preventive measures may be needed to guard against sexually transmitted diseases or pregnancy. A professionally conducted medical history and examination are essential for both the victim’s future health and potential legal proceedings. However, this can only be achieved with accepted guidelines, ongoing education, and further training of health care professionals.

## Figures and Tables

**Table 1:** Recommended questions for the parent [7,11].

Subject	Questions
Alarm	“What made you think your child had been abused?”
Timeline	“How did it start?” “When was the last time your child was with the person you believe may have abused them?”
Physical signs	“Does your child have any symptoms?” “Are there any problems with urination or defecation?”
Behavioral signs	“Have you noticed any changes in their behaviour?”
Medical history	“Has your child ever experienced trauma or injury to their genitals?” “Has your child ever been abused?”
Social history	“Who else lives at home and who takes care of the child?”

**Table 2:** Advanced communication with the victim [5,7,11].

Suggested Questions	Avoid
“What is your favourite hobby?”	“Do you like playing tennis?”
“Tell me why you’re here today”.	“Do you know why you are here?”
“How do you call where you pee? Where does poop come out?”	“Has anyone ever touched you there in a way you didn’t like?”
“Tell me about everyone who has touched your private parts”.	“Did he touch your private parts?”
“Where were your clothes when this happened?”	“Were your clothes off?”
“What questions do you want to ask?”	“Do you have any questions?”

**Table 3:** Aims of medical examination.

Confirm suspicion of child sexual abuse (CSA)
Provide immediate care: diagnosis and treatment of injuries
Offer prompt feedback to parents about the events involving their child
Reassure the family and the child about the quick improvement of the child’s health
Conduct screening and prophylaxis for sexually transmitted infections (STIs)
Perform pregnancy screening
Administer emergency contraception if needed
Determine necessary further steps: involvement of police, psychologist, psychiatrist, or guardian
Collect trace evidence for future forensic analysis
Provide accurate documentation for further legal proceedings

**Table 4:** Timing of medical examination [5].

Emergency examination	Immediate care for acute pain, bleeding, or suicidal ideation
	Alleged assault within the previous 72 h: collection of evidence for later forensic analysis
	Provision of emergency contraception if applicable
	Administer STI prophylaxis if needed
Urgent examination	Sexual contact within the past 2 weeks without immediate care required
Non-urgent examination	Sexual contact occurred more than 2 weeks prior

**Table 7:** Abridged Adams Classification [5,19].

Type	Explanation
Adams I	Physiological examination findings. No suspicion of abuse. The physical findings of anogenital injury are consistent with a history of an accident.
Adams II	Medical findings of unclear significance. Suspicion of CSA cannot be ruled out.
Adams III	Pathological examination findings. The findings indicate a diagnosis of CSA. Clear signs of penetration and violence without a history of accident.
